# Genetic analysis of seedling root traits reveals the association of root trait with other agronomic traits in maize

**DOI:** 10.1186/s12870-018-1383-5

**Published:** 2018-08-15

**Authors:** Chuanli Ju, Wei Zhang, Ya Liu, Yufeng Gao, Xiaofan Wang, Jianbing Yan, Xiaohong Yang, Jiansheng Li

**Affiliations:** 1 College of Life Sciences, Capital Normal University, Beijing, 100048 China; 2 National Maize Improvement Center of China, Beijing Key Laboratory of Crop Genetic Improvement, China Agricultural University, Beijing, 100193 China; 3 Maize Research Center, Beijing Academy of Agricultural and Forestry Sciences, Beijing, 100097 China; 4 National Key Laboratory of Crop Genetic Improvement, Huazhong Agricultural University, Wuhan, 430070 China

**Keywords:** Maize, Seedling root traits, QTL mapping, Genetic association, Agronomic traits

## Abstract

**Background:**

Root systems play important roles in crop growth and stress responses. Although genetic mechanism of root traits in maize (*Zea mays* L.) has been investigated in different mapping populations, root traits have rarely been utilized in breeding programs. Elucidation of the genetic basis of maize root traits and, more importantly, their connection to other agronomic trait(s), such as grain yield, may facilitate root trait manipulation and maize germplasm improvement. In this study, we analyzed genome-wide genetic loci for maize seedling root traits at three time-points after seed germination to identify chromosomal regions responsible for both seedling root traits and other agronomic traits in a recombinant inbred line (RIL) population (Zong3 × Yu87–1).

**Results:**

Eight seedling root traits were examined at 4, 9, and 14 days after seed germination, and thirty-six putative quantitative trait loci (QTLs), accounting for 9.0–23.2% of the phenotypic variation in root traits, were detected. Co-localization of root trait QTLs was observed at, but not between, the three time-points. We identified strong or moderate correlations between root traits controlled by each co-localized QTL region. Furthermore, we identified an overlap in the QTL locations of seedling root traits examined here and six other traits reported previously in the same RIL population, including grain yield-related traits, plant height-related traits, and traits in relation to stress responses. Maize chromosomal bins 1.02–1.03, 1.07, 2.06–2.07, 5.05, 7.02–7.03, 9.04, and 10.06 were identified QTL hotspots for three or four more traits in addition to seedling root traits.

**Conclusions:**

Our identification of co-localization of root trait QTLs at, but not between, each of the three time-points suggests that maize seedling root traits are regulated by different sets of pleiotropic-effect QTLs at different developmental stages. Furthermore, the identification of QTL hotspots suggests the genetic association of seedling root traits with several other traits and reveals maize chromosomal regions valuable for marker-assisted selection to improve root systems and other agronomic traits simultaneously.

**Electronic supplementary material:**

The online version of this article (10.1186/s12870-018-1383-5) contains supplementary material, which is available to authorized users.

## Background

The root system, which plays an important role in crop growth and stress responses, anchors the shoots, absorbs water and nutrients from the soil, and is the biosynthetic site of phytohormones required for plant development [1]. As the site of plant-soil interactions, the roots also play a key role in plant responses to environmental changes and influence agronomically important traits, such as drought or flood tolerance [2–4], root-lodging resistance [5, 6], and the efficiency of nutrient use under suboptimal growth conditions [7–10]. The effectiveness of root function depends on the characteristics and architecture of the root system.

Maize (*Zea mays* L.) has a complex root system consisting of embryonic and post-embryonic roots. The embryonic root system is composed of a primary root and several seminal roots, while the post-embryonic root system includes crown roots, brace roots, and lateral roots generated from all the former root types [11]. A single primary root (> 1 cm) emerges from the seed at approximately 3 days after germination (dag), and a variable number of seminal roots start to appear at about 5 dag [11]. The development of the post-embryonic root system can be divided into two stages. During the early stage, lateral roots start to appear on the primary and seminal roots at approximately 10 dag, and crown roots initiate about 2 weeks after germination. The late stage starts approximately 3 to 4 weeks after germination when whorls of crown roots form at the second node, and brace roots are also developed [11]. The embryonic root system is critically involved in seedling vigor [12] and functions throughout the life cycle of the plant [11, 13]. The post-embryonic lateral roots are the major site of water and mineral uptake [14, 15], while the post-embryonic crown roots are the basis for plant lodging resistance [11]. Because mature plants have large and complex root systems, young maize seedlings are often used for root trait analysis [11]. For example, as well as being small in size, 2-week-old seedlings have embryonic primary and seminal roots as well as early post-embryonic lateral and crown roots, i.e., they possess all the root types present in mature plants, with the exception of brace roots [12].

Maize root traits can be regulated by monogenes [11], such as *RTCS* for seminal and shoot-borne root initiation [16, 17], *RTH1, RTH2,* and *RTH3* for root hair elongation [11, 18, 19], and *RUM1* for seminal and lateral root initiation [20]; however, some root features are inherited quantitatively and controlled by many genes [1, 7]. Quantitative trait locus (QTL) mapping of maize root traits, such as primary root length (PRL), total root length (TRL), seminal root number, lateral root number (LRN), and seedling root dry weight, has been conducted in different linkage populations, and a variety of root trait QTLs have been reported [21–27]. Despite the availability of a great deal of genetic information, root traits have seldom been utilized as selection criteria in maize breeding programs because of the difficulty in examining the field traits non-destructively. An improved knowledge of the genetic architecture of maize root traits and, more importantly, knowledge of their genetic connection to other trait(s) may facilitate better implementation of marker-assisted selection (MAS) to improve maize yields.

Correct phenotyping is an essential prerequisite for accurate QTL mapping of root traits. Different root phenotyping methods have been developed in maize, with plant cultivation settings ranging from the field, to the greenhouse, and the controlled growth chamber [1, 16, 21–25, 28–30]. For studies performed in the field or a greenhouse with soil-containing pots [22, 29], root traits are evaluated in soil-grown plants to provide information in an agriculturally relevant context. However, the soil properties of different fields may vary, and high-throughput extraction of intact root systems from the soil is laborious and technically challenging. Studies of plants cultivated in hydroponic or gellan gum-based media in a growth chamber [21, 23, 24, 26, 27] offer precise conditions for root growth and are convenient for subsequent data collection, although the phenotypic relevance of these non-soil methods to soil-grown plants is a major concern because root architecture is plastic and very susceptible to environmental changes [1, 31–33]. Overall, plant cultivation methods that closely mimic the soil medium, are stable in nutrient and environmental factors, and are easy to operate, are suitable for maize root trait phenotyping.

In this study, we used a recombinant inbred line (RIL) population that has been investigated for the genetic mechanisms of several other traits to (1) identify QTLs for maize seedling root traits at 4, 9, and 14 dag; (2) determine QTL locations at, and between, each time-point; (3) discover chromosomal regions affecting both root traits and other agronomic traits, such as grain yield and those closely related to roots.

## Results

### Phenotypic variation in seedling root traits

**Fig. 1:**
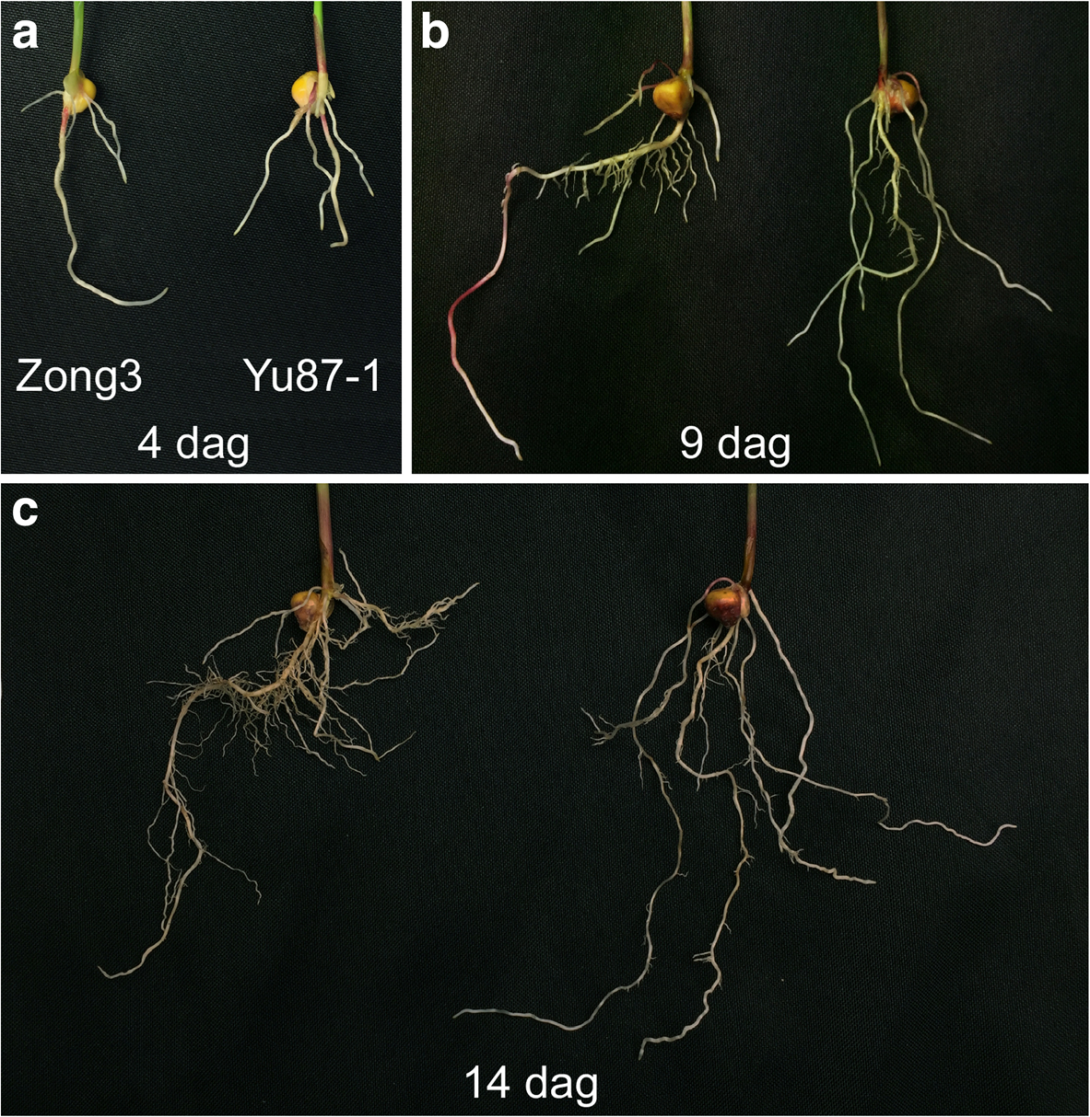
Root phenotypes of the RIL parental lines Zong3 and Yu87–1 in seedlings at 4 dag (**a**), 9 dag (**b**), and 14 dag (**c**). Representative roots are shown in each image, with Zong3 on the left and Yu87–1 on the right

The maize seedlings were grown in fine-grained quartz sand in a growth chamber for root trait phenotyping. As shown in Fig. 1, the two parental lines of the RIL population, Zong3 and Yu87–1, showed clear differences in root morphology in seedlings at 4 dag, 9 dag, and 14 dag. Zong3 possessed a longer, thicker primary root than that of Yu87–1 from 4 dag (Fig. 1a), in addition to more numerous and longer lateral roots on the primary root than Yu87–1 in seedlings at 9 dag and 14 dag (Fig. 1b and c). Yu87–1 was superior to Zong3 in that it tended to have a greater total number of the primary, seminal, and crown roots (PSC) (Fig. 1). We further assessed the architectural differences between the two root systems in terms of eight quantified traits (Table 1): PRL, PSC, LRN, total root tip number (RTN), TRL, total root surface area (RSA), total root volume (TRV), and average root diameter (ARD). Five of the quantified root traits listed were obtained from scanned images (Table 1); an representative image is shown (Additional file 1: Figure S1). Analysis of the data of the parental lines (Additional file 2: Table S1) revealed progressive increases in seven of the eight root traits over the three time-points, while the remaining trait, ARD, decreased gradually (Table 2), presumably due to the continuous addition of new and fine roots over time. This result shows the rapid development of both seedling root systems with the extension of time, which supports the relevance of the three experimental time-points chosen for root trait evaluation in this study. In accordance with the morphological differences between the two root systems (Fig. 1), there were significant differences in the data for 21 of the total of 24 quantified root traits between two parental lines (Table 2; *P* < 0.05 or *P* < 0.01). The LRN, RTN, TRL, and RSA values for Zong3 seedlings were significantly higher than those of Yu87–1 at both 9 dag and 14 dag (Table 2; *P* < 0.01). In most cases, the root traits of Zong3 were superior to those of Yu87–1, particularly at later time-points (Table 2). For example, in 14 dag seedlings, the traits LRN, RTN, TRL, and RSA in Zong3 versus Yu87–1 were 123.67 and 49.67, 135.07 and 62.53, 178.89 cm and 99.86 cm, and 50.91 cm^2^ and 31.22 cm^2^, respectively (Table 2). Yu87–1 showed superiority over Zong3 in terms of PSC at all three time-points and RTN at 4 dag (Table 2; *P* < 0.01). Taken together, these results show that the two parental lines displayed significant differences in seedling root traits at all three of the time-points evaluated, and that the two lines can be distinguished effectively by quantitative measurement of eight seedling root traits.

**Table 1:** Eight seedling root traits analyzed in this study

Root trait	Abbreviation	Measurement description
Primary root length	PRL	Measured using a ruler
Total number of the primary, seminal, and crown roots	PSC	Counted manually
Lateral root number	LRN	Calculated by subtracting PSC from RTN
Total root tip number	RTN	Measured electronically
Total root length	TRL	Measured electronically
Total root surface area	RSA	Measured electronically
Total root volume	TRV	Measured electronically
Average root diameter	ARD	Measured electronically

**Table 2:** Seedling root traits in the two parental lines and the RIL population

Root trait	Time^a^	Zong3	Yu87–1	Significance	RILs		*h*^*2*^(%)^c^
	(dag)	Mean	Mean	level^b^	Mean	Range	
PRL (cm)	4	7.80	5.94	**	8.53	4.89–12.43	87.6
	9	15.96	14.94	*	17.55	11.73–22.30	84.2
	14	20.40	17.17	**	21.95	16.82–28.63	75.5
PSC	4	3.07	4.27	**	4.21	1.83–7.67	88.8
	9	5.80	7.73	**	8.58	3.82–13.67	83.6
	14	11.40	12.80	**	9.75	5.50–14.75	83.1
LRN	4	3.60	4.53	NS	7.51	0.39–17.06	87.8
	9	46.87	34.87	**	60.56	22.04–166.26	89.4
	14	123.67	49.67	**	104.44	41.00–187.75	91.3
RTN	4	6.67	9.00	**	11.72	3.73–21.6	90.2
	9	52.60	42.60	**	69.14	26.44–174.90	89.9
	14	135.07	62.53	**	114.19	50.6–198.2	91.6
TRL (cm)	4	14.85	14.31	NS	21.56	6.86–37.17	91.3
	9	66.20	53.03	**	90.51	42.16–156.88	91.1
	14	178.89	99.86	**	160.31	76.91–244.25	93.3
RSA (cm^2^)	4	6.48	5.44	NS	6.08	1.93–11.57	92.9
	9	21.08	18.10	*	29.98	11.15–54.31	92.6
	14	50.91	31.22	**	55.14	22.39–91.64	91.9
TRV (cm^3^)	4	0.29	0.16	**	0.14	0.04–0.26	89.9
	9	0.54	0.42	*	0.81	0.22–1.71	90.6
	14	1.12	0.84	**	1.53	0.52–2.77	87.9
ARD (mm)	4	1.45	1.19	**	0.90	0.57–1.20	87.9
	9	0.98	1.05	*	1.06	0.73–1.38	89.9
	14	0.88	1.05	**	1.08	0.79–1.34	90.2

^a^The time-points for seedling root trait evaluation: 4 days after germination (dag), 9 dag, and 14 dag

^b^The level of statistical significance of differences between two maize genotypes analyzed by *t*-test: NS, not significant; *, significant at *P* < 0.05; **, significant at *P* < 0.01

^c^Broad-sense heritability detected

We next analyzed the root traits in the Zong3 × Yu87–1 RIL population. For each root trait, we detected a broad spectrum of phenotypic variation (Table 2; Additional file 3: Table S2) and an approximately normal distribution (Additional file 4: Figure S2; Additional file 5: Table S3). The mean values of sixteen out of a total of twenty four sets of root trait data in the RIL population were higher than those in both parental lines (Table 2). The broad-sense heritability (*h*^*2*^) of root traits ranged from 75.5 to 93.3% (Table 2), which was comparable to the yield trait results (77.4–88.2%) [34] and slightly higher than the results for the other two traits (67–87%) detected in the same RIL population [35, 36]. The high heritability values indicate that the phenotypic variations in root traits are controlled predominantly by genetics in comparison to the minor effect of environmental factors.

We next performed Pearson correlation analysis to investigate the phenotypic relationships among the root traits at each time-point. As shown in Table 3, similar patterns of correlations were observed at all three time-points, with more significant correlations detected at the later time-points. For example, the correlation coefficients between PRL, PSC, LRN, RTN, or ARD and other traits were similar at all three time-points, and each trait was correlated more significantly with other traits at 14 dag than at 4 dag (Table 3; *P* < 0.05 or *P* < 0.01). Among the eight seedling root traits, RSA and TRV exhibited significant correlations with the other seven traits at all three time-points, and TRL correlated with all other traits except ARD (Table 3; *P* < 0.01). Notably, strong associations (*r* > 0.900) were detected between LRN and RTN at all time-points (Table 3; *P* < 0.01), which confirmed the predominant contribution of LRN to RTN. Strong associations (*r* > 0.900) were also identified between RSA and TRV at all time-points (Table 3; *P* < 0.01). Moderate correlations existed at all time-points (0.554 < *r* < 0.941) between TRL and the traits, RTN, RSA, and TRV (Table 3; *P* < 0.01). ARD correlated significantly with only RSA and TRV, in seedlings at 4 dag and 9 dag (Table 3; 0.353 < *r* < 0.635; *P* < 0.01), and there were additional weak correlations with PSC, LRN, and RTN in seedlings at 14 dag (Table 3; 0.218 < *r* < 0.270; *P* < 0.05 or *P* < 0.01). PRL shared no significant correlations with PSC and ARD at any of the time-points (Table 3).

**Table 3:** Pearson correlation coefficients among seedling root traits at each time-point after germination

	PRL	PSC	LRN	RTN	TRL	RSA	TRV
4 dag							
PSC	− 0.150						
LRN	0.099	0.236*					
RTN	0.054	0.459**	0.971**				
TRL	0.629**	0.409**	0.497**	0.554**			
RSA	0.609**	0.381**	0.402**	0.460**	0.941**		
TRV	0.517**	0.354**	0.262**	0.326**	0.814**	0.942**	
ARD	0.112	0.011	−0.156	−0.140	0.122	0.418**	0.618**
9 dag							
PSC	−0.182						
LRN	0.562**	0.184					
RTN	0.536**	0.266**	0.997**				
TRL	0.525**	0.428**	0.791**	0.812**			
RSA	0.431**	0.401**	0.798**	0.817**	0.884**		
TRV	0.306**	0.323**	0.700**	0.714**	0.678**	0.941**	
ARD	−0.147	0.037	0.112	0.113	− 0.105	0.353**	0.635**
14 dag							
PSC	−0.059						
LRN	0.605**	0.236*					
RTN	0.593**	0.289**	0.998**				
TRL	0.648**	0.340**	0.788**	0.795**			
RSA	0.552**	0.381**	0.744**	0.754**	0.888**		
TRV	0.429**	0.377**	0.660**	0.671**	0.729**	0.957**	
ARD	0.083	0.218*	0.261**	0.270**	0.192	0.603**	0.789**

Correlation coefficients were analyzed among root traits detected on the same day, and values greater than 0.90 are highlighted in italics. The symbol * or ** indicates that the correlation between root traits is statistically significant at the level of 0.05 (*P* < 0.05) or 0.01 (*P* < 0.01), respectively

The correlations for each root trait were also determined at each time-point. PRL, PSC, TRL, RSA, TRV, and ARD were significantly correlated at the three time-points, with correlation coefficients ranging between 0.272 and 0.730 (Table 4; *P* < 0.01). LRN and RTN were significantly correlated at 9 dag and 14 dag, and not statistical at 4 dag (Table 4).

**Table 4:** Pearson correlation coefficients for seedling root traits at each time-point after germination

	4 dag	9 dag
PRL		
9 dag	0.629^**^	
14 dag	0.570^**^	0.730^**^
PSC		
9 dag	0.504^**^	
14 dag	0.521^**^	0.700^**^
LRN		
9 dag	0.129	
14 dag	0.028	0.532^**^
RTN		
9 dag	0.165	
14 dag	0.071	0.535^**^
TRL		
9 dag	0.421^**^	
14 dag	0.272^**^	0.623^**^
RSA		
9 dag	0.481^**^	
14 dag	0.318^**^	0.539^**^
TRV		
9 dag	0.456^**^	
14 dag	0.320^**^	0.477^**^
ARD		
9 dag	0.424^**^	
14 dag	0.334^**^	0.337^**^

The symbol ** indicates that the correlation for the root trait between different time-points is statistically significant at the 0.01 level (*P* < 0.01)

### Detection of QTLs for seedling root traits

**Fig. 2:**
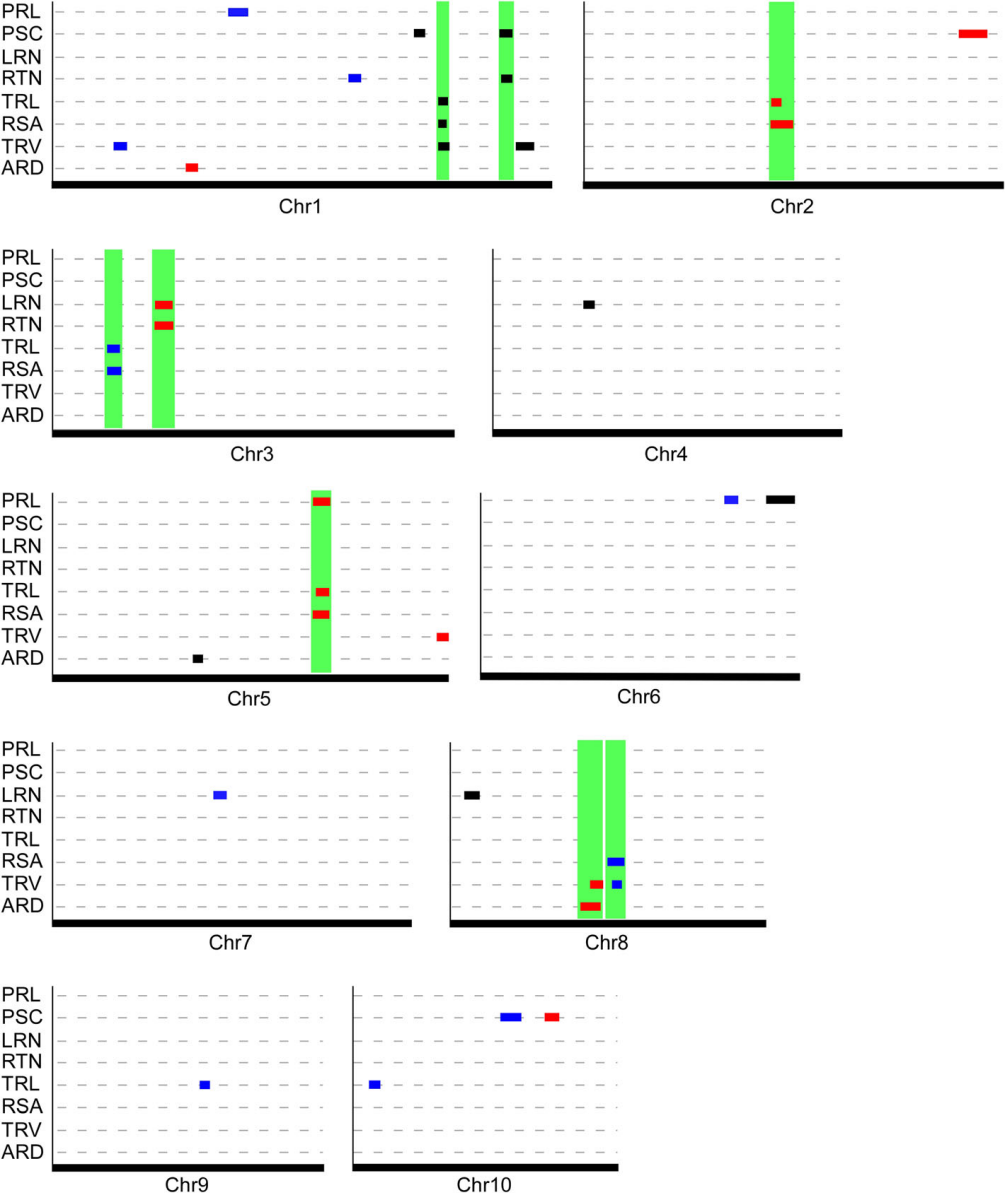
QTL plots for eight seedling root traits across three time-points after germination identified in the Zong3 × Yu87–1 RIL population. In each small plot, the x-axis represents the indicated maize chromosome, with the length proportional to that of the genetic linkage map, and the y-axis lists the eight seedling root traits analyzed. QTLs are shown as colored boxes on the relative positions of the chromosomes, with QTLs detected at 4 dag (black), 9 dag (blue), and 14 dag (red). The green rectangles show the chromosomal regions in which the different QTLs co-localized

Based on a high-density genetic linkage map consisting of 3184 bins [37], we detected a total of thirty-six putative QTLs for eight seedling root traits across the three time-points (Tables 5 and 6). The logarithm of odds (LOD) threshold values were between 3.3 and 3.7 according to permutation tests (1000 times) (Table 5). These QTLs were distributed on all 10 chromosomes, with chromosomes 1, 5, and 8 each containing at least five QTLs (Fig. 2; Table 6). The individual QTLs explained between 8.9 and 23.2% of the phenotypic variation (Table 6). Similar numbers of QTLs were detected at each time-point, with eleven at 4 dag, twelve at 9 dag, and thirteen at 14 dag (Table 5). Among these QTLs, the alleles that are associated with increased root traits at twenty loci were contributed by parental line Zong3, and the alleles that are associated with increased root traits at sixteen loci were contributed by Yu87–1 (Table 6), suggesting the essential roles of both parental lines in root trait determination in the RIL population.

**Table 5:** The number of putative QTLs detected

Root trait	4 dag		9 dag		14 dag		QTL No. at 3 time-points
	LOD	QTL No.	LOD	QTL No.	LOD	QTL No.	
PRL	3.6	1	3.5	2	3.4	1	4
PSC	3.5	2	3.5	1	3.4	2	5
LRN	3.5	2	3.4	1	3.6	1	4
RTN	3.5	1	3.3	1	3.7	1	4
TRL	3.5	1	3.5	3	3.5	2	6
RSA	3.6	1	3.6	2	3.7	2	5
TRV	3.5	2	3.5	2	3.5	2	6
ARD	3.4	1		0	3.6	2	3
QTL No.		11		12		13	36

LOD represents the logarithm of odds threshold for each root trait

**Table 6:** Summary of root trait QTLs detected in the Zong3 × Yu87–1 RIL population

QTL^a^	Chr	Flanking markers	Genetic interval (cM)	Maximum LOD	A^b^	R^2^(%)^c^	Bin^d^
4 dag							
_*4d*_*PRL6–1*	6	SYN13318–SYN38609	202.9–218.1	3.7	− 0.49	10.7	6.07
_*4d*_*PSC1–1*	1	SYN34256–PZE-101206740	274.3–276.7	3.6	0.29	9.2	1.02
_*4d*_*PSC1–2*	1	SYN19016–SYN3286	338.7–343.0	3.9	0.30	10.0	1.02
_*4d*_*LRN4–1*	4	PZE-104018915–PZE-104021108	71.4–74.7	5.9	1.41	16.7	4.03
_*4d*_*LRN8–1*	8	PZE-108002915–SYN27442	15.1–21.1	3.8	1.12	10.6	8.01
_*4d*_*RTN1–1*	1	SYN33506–SYN3286	340.0–343.0	4.8	1.33	13.4	1.02
_*4d*_*TRL1–1*	1	PZE-101212333–PZE-101213906	292.0–293.9	5.5	2.35	16.0	1.02
_*4d*_*RSA1–1*	1	PZE-101212333–PZE-101213812	292.0–293.4	6.0	0.73	15.7	1.02
_*4d*_*TRV1–1*	1	PZE-101212333– PZE-101215284	292.0–296.1	5.3	0.02	13.6	1.02
_*4d*_*TRV1–2*	1	SYN29074–PZA01238.2	346.6–355.6	3.6	0.02	9.0	1.03
_*4d*_*ARD5–1*	5	PZA-000996001–SYN26430	100.9–102.5	8.5	−0.06	23.2	5.03
9 dag							
_*9d*_*PRL1–1*	1	PZE-101120159–PZA01267.3	133.8–143.0	3.6	0.72	10.0	1.03
_*9d*_*PRL6–1*	6	PZA00223.4–PHM597.12	173.0–177.9	6.6	1.21	19.8	6.06
_*9d*_*PSC10–1*	10	PZE-110074413–SYN22956	113.5–124.6	4.0	0.61	9.7	10.05
_*9d*_*LRN7–1*	7	PZE-107068924–PZE-107073348	122.9–127.8	4.1	7.53	11.9	7.02–7.03
_*9d*_*RTN1–1*	1	PZE-101171899–SYN31725	222.7–226.6	3.7	−7.45	10.2	1.07
_*9d*_*TRL3–1*	3	PZE-103012466–PZE-103013845	44.3–48.2	4.8	−7.33	11.8	3.02
_*9d*_*TRL9–1*	9	SYN22542–PUT-163a-60,342,651–2460	113.1–115.5	3.7	6.73	8.9	9.04
_*9d*_*TRL10–1*	10	SYN12403–PUT-163a-148,991,534-709	15.5–18.9	5.9	−8.49	14.9	10.01
_*9d*_*RSA3–1*	3	PZE-103012466–PZE-103014951	44.3–49.8	4.0	−2.41	10.4	3.02
_*9d*_*RSA8–1*	8	PZE-108075125–PZE-108083525	119.4–127.2	3.8	−2.37	10.0	8.05
_*9d*_*TRV1–1*	1	PZE-101024131–SYN10530	47.7–52.9	4.4	−0.08	12.0	1.02
_*9d*_*TRV8–1*	8	SYN20177–SYN23195	123.7–126.3	5.6	−0.10	15.6	8.05
14 dag							
_*14d*_*PRL5–1*	5	PZE-105130621–PZE-105133858	185.4–191.9	4.4	−0.86	13.0	5.05
_*14d*_*PSC2–1*	2	ZM015502–0192–PZE-102189664	283.0–298.8	4.8	−0.63	12.7	2.09
_*14d*_*PSC10–1*	10	PZE-110091124–PZE-110093416	146.4–151.5	4.6	0.59	11.7	10.06
_*14d*_*LRN3–1*	3	PZE-103030194–ZM013524–0806	79.9–87.7	3.7	11.29	10.1	3.04
_*14d*_*RTN3–1*	3	PZE-103030194–ZM013524–0806	79.9–87.7	3.6	11.31	9.9	3.04
_*14d*_*TRL2–1*	2	PZE-102107608–SYN36647	143.4–144.5	6.1	13.64	16.4	2.05
_*14d*_*TRL5–1*	5	PZA02788.12–SYN35856	186.9–190.9	5.2	−11.94	13.9	5.05
_*14d*_*RSA2–1*	2	SYN27899–PZE-102120088	142.7–154.7	5.4	5.58	15.8	2.06–2.07
_*14d*_*RSA5–1*	5	PZE-105129694–PZE-105132581	184.6–191.1	3.8	−4.31	10.5	5.05
_*14d*_*TRV5–1*	5	SYN33305–PZE-105181352	271.3–273.5	4.3	0.18	12.2	5.09
_*14d*_*TRV8–1*	8	PZE-108070036–PZE-108073190	110.8–115.5	6.0	−0.21	17.7	8.05
_*14d*_ *ARD1–1*	1	PZE-101056856–PZE-101061035	102.0–105.4	3.7	−0.04	10.6	1.03
_*14d*_*ARD8–1*	8	PZE-108064797–PZE-108072092	104.0–113.1	6.0	−0.05	17.5	8.04–8.05

^a^The QTLs detected; the name includes information of seedling cultivation time, trait abbreviation, and chromosome number

^b^The additive effect of putative QTLs, with positive values indicating that alleles from Zong3 increased the trait and negative values indicating that alleles from Yu87–1 increased the trait

^c^The percentage of phenotypic variation each putative QTL

^d^The bin positions of the QTLs, based on the B73 physical map from the website (http://www.maizegdb.org)

Subsequent analysis of the root trait QTLs identified at each time-point revealed QTL co-localizations. Seven of a total of eleven QTLs identified at 4 dag were localized on chromosome 1, while the other four were localized on additional different chromosomes (Fig. 2; Table 6). The seven QTLs localized on chromosome 1 spanned four regions (Fig. 2), with _*4d*_*TRL1–1*, _*4d*_*RSA1–1*, and _*4d*_*TRV1–1* co-localizing at approximately 292–293 centimorgan (cM) genetic interval, while _*4d*_*PSC1–2* and _*4d*_*RTN1–1* co-localizing at approximately the 340–343 cM region, and _*4d*_*PSC1–1* and _*4d*_*TRV1–2* localizing at the 274.3–276.7 cM and 346.6–355.6 cM regions, respectively (Table 6). The QTLs detected at 4 dag each contributed between 9.0 and 23.2% of the phenotypic variation in root traits, with the one controlling ARD (_*4d*_*ARD5–1*, LOD = 8.5, R^2^ = 23.2%) localized on chromosome 5 being the most significant (Table 6). Among the QTLs identified at 9 dag, four co-localized in two chromosomal regions, with _*9d*_*TRL3–1* and _*9d*_*RSA3–1* at the 44.3–48.2 cM region on chromosome 3 and _*9d*_*RSA8–1* and _*9d*_*TRV8–1* at the 123.7–126.3 cM region on chromosome 8; the remaining eight QTLs were non-overlapped and distributed in various regions of five chromosomes (Fig. 2; Table 6). The largest effect at 9 dag was contributed by the QTL controlling PRL located on chromosome 6 (_*9d*_*PRL6–1*, LOD = 6.6, R^2^ = 19.8%) which had alleles from Zong3 that increased root traits (Table 6). Among the QTLs identified at 14 dag, nine were clustered in four overlapping chromosomal regions, with _*14d*_*TRL2–1* and _*14d*_*RSA2–1* co-localizing on chromosome 2, _*14d*_*LRN3–1 and*_*14d*_*RTN3–1* co-localizing on chromosome 3, _*14d*_*PRL5–1*, _*14d*_*TRL5–1*, and _*14d*_*RSA5–1* co-localizing on chromosome 5, and _*14d*_*TRV8–1* and _*14d*_*ARD8–1* co-localizing on chromosome 8, while the remaining four QTLs were localized on additional different chromosomes (Fig. 2; Table 6). Notably, the two QTLs co-localized on chromosome 8, _*14d*_*TRV8–1* and _*14d*_*ARD8–1*, both had large effects (R^2^ = 17.7% and R^2^ = 17.5%, respectively) and both had alleles that increased root traits from Yu87–1 (Table 6). In summary, we detected two co-localized QTL regions on chromosome 1 that controlled root traits at 4 dag, two co-localized QTL regions controlling root traits at 9 dag, with one on chromosome 3 and the other one on chromosome 8, and four co-localized QTL regions located on chromosomes 2, 3, 5, and 8, respectively, that controlled root traits at 14 dag (Fig. 2; Table 6).

Our further analysis showed that root traits controlled by each co-localized QTL region were strongly or moderately correlated to each other. For example, the correlation coefficients among root traits (TRL, RSA, and TRV) controlled by one co-localized QTL region at 4 dag were 0.814, 0.941, and 0.942, respectively, while the correlation coefficient between traits (PSC and RTN) controlled by the other co-localized QTL region at 4 dag was 0.459 (Table 3; Fig. 2). In terms of the situation at 9 dag, the correlation coefficient between root traits (TRL and RSA) controlled by one co-localized QTL region was 0.884, and the correlation coefficient between traits (RSA and TRV) controlled by the other co-localized QTL region was 0.941 (Table 3; Fig. 2). As for the situation at 14 dag, the correlation coefficients between or among root traits controlled by each of the four co-localized QTL regions ranged between 0.552 and 0.998, with the correlation coefficient between traits LRN and RTN being the highest (Table 3).

We next determined the locations of root trait QTLs among the different time-points. For all the QTLs identified (eleven at 4 dag, twelve at 9 dag, and thirteen at 14 dag), we found no overlapping loci that controlled the same root traits among the three time-points. Nor did we find overlapping loci that controlled different traits among the time-points (Fig. 2). Two QTLs detected at 9 dag appeared to be localized close to two QTLs detected at 14 dag on chromosome 8 (Fig. 2); however, there was no overlap according to the genetic intervals determined. The two QTLs detected at 9 dag were localized at 119.4–127.2 cM (for _*9d*_*RSA8–1*) and 123.7–126.3 cM (for _*9d*_*TRV8–1*), yet the two QTLs detected at 14 dag were localized at 104.0–113.1 cM (for _*14d*_*RAD8–1*) and 110.8–115.5 cM (for _*14d*_*TRV8–1*), respectively (Table 6). Therefore, although co-localization of root trait QTLs was detected at each time-point, no co-localizations of root trait QTLs were found among the different time-points.

We also analyzed the number of QTL parental alleles that increased root traits. Despite the similar number of root trait QTLs identified at each time-point (Table 5), the alleles responsible for increasing the root traits did not originate evenly from the two parental lines. For the eleven QTLs detected at 4 dag, the alleles that increased root traits at nine loci were contributed by Zong3, and the alleles that increased root traits at the other two loci were contributed by Yu87–1 (Table 6). For the twelve QTLs identified at 9 dag, the alleles that increased root traits at five loci were from Zong3, and the alleles at the other seven loci were from Yu87–1 (Table 6). In terms of the thirteen QTLs detected at 14 dag, the alleles that increased root traits at six loci were contributed by Zong3, and the alleles at seven loci were contributed by Yu87–1 (Table 6). Therefore, the proportion of favorable QTL alleles derived from the two parental lines changed with the extension of time, with the number of favorable alleles from Zong3 gradually decreasing and the number of favorable alleles from Yu87–1 increasing.

### Identification of chromosomal regions containing QTLs for seedling root traits co-localized with QTLs for other traits

The genetic basis of several traits have been investigated in the Zong3 × Yu87–1 RIL population, including plant height-related traits [38, 39], the amount of toxic metalloid arsenic (As) in above-ground tissues [40], grain yield-related traits [34, 41], root traits under hydroponic low nitrogen (LN) or high nitrogen (HN) conditions [42], several physiological traits associated with drought stress [36], and disease resistance [35]. Therefore, in this study, we next identified common chromosomal regions that control seedling root traits examined in this study as well as the listed traits previously identified on the same genetic background. The QTLs identified in the current and previous studies were compared on the basis of the bin locations of the single nucleotide polymorphism (SNP) and simple sequence repeat (SSR) molecular markers flanking the QTLs on the maize genome (http://www.maizegdb.org). Multiple co-localized chromosomal regions at the bin level that co-controlled seedling root traits and other traits were identified (Table 7).

**Table 7:** Summary of chromosomal regions with co-localized QTLs for seedling root traits identified in this study and QTLs for six other traits reported previously in the same RIL population

QTLs identified in this study		QTLs reported in previous studies			
QTL	Bin	Trait^a^	Marker interval	Bin	Reference
_*4d*_*PSC1–1*, _*4d*_*PSC1–2*, _*4d*_*RTN1–1*, _*4d*_*TRL1–1,*_*4d*_*RSA1–1*, _*4d*_*TRV1–1*, _*9d*_*TRV1–1*	1.02	As content in stem	phi001–bnlg1083	1.02–1.03	[40]
		Internode number	phi427913–phi001	1.02	[38]
		MARL in HN	phi427913–bnlg1614	1.01–1.02	[42]
_*4d*_*TRV1–2*, _*9d*_*PRL1–1,*_*14d*_ *ARD1–1*	1.03	Grain yield, Row number	bnlg2180–umc1169	1.03–1.04	[34]
		Internode number	phi001–bnlg1484	1.03	[38]
		ARL in LN/HN	bnlg1866–bnlg2180	1.03	[42]
_*9d*_ *RTN1–1*	1.07	Plant height	umc2151–bnlg1556	1.07	[38]
		Leaf number	umc1335–umc1122	1.07	[38]
		Internode number	umc2151–umc1122	1.07	[38]
		MARL in LN, AARL in LN	bnlg1025–umc2029	1.07–1.09	[42]
		RSFW, RSDW	bnlg1556–umc2029	1.07–1.08	[36]
_*14d*_ *RSA2–1*	2.06–2.07	As content in stem	bnlg1633–umc1042	2.07	[40]
		Plant height	umc2372–umc1497	2.07	[38]
		AARL in LN	bnlg1633–umc1042	2.07	[42]
		RSFW, RSDW, LTD	umc1042–bnlg2144	2.07–2.08	[36]
_*14d*_*LRN3–1*, _*14d*_*RTN3–1*	3.04	Leaf number	umc1223–umc1773	3.04	[38]
		Leaf number	umc2002–bnlg1035	3.04	[38]
		Average internode length	umc1773–bnlg1035	3.04	[38]
		ARN in LN/HN	umc1504–umc1523	3.04	[42]
*4dLRN4–1*	4.03	Grain yield	umc2082–umc2176	4.03	[34]
_*4d*_ *ARD5–1*	5.03	Plant height, Average internode length	umc1692–umc2373	5.03	[38]
		Resistance to common smut	phi109188–umc2373	5.03	[35]
_*14d*_*PRL5–1*, _*14d*_*TRL5–1*, _*14d*_*RSA5–1*	5.05	Ear length Plant height	umc1019–bnlg1237 umc1155–bnlg1237	5.05–5.06 5.05	[34] [38]
		Average internode length	umc1155–umc1019	5.05	[38]
		LRL in LN/HN	bnlg278–phi048	5.05–5.07	[42]
_*14d*_ *TRV5–1*	5.09	AARL in LN/HN	umc1792–umc1829	5.08–5.09	[42]
_*4d*_ *PRL6–1*	6.07	Internode number	umc1020–phi299852	6.07	[38]
		AARL in LN/HN	umc1063–phi299852	6.07	[42]
_*9d*_ *LRN7–1*	7.02–7.03	Ear length	bnlg1792–umc2142	7.02	[34]
		Grain yield	bnlg339–umc1865	7.03	[34]
		As content in leaves	umc2142–mmc0411	7.02–7.03	[40]
		Internode number	blg1792–mmc0411	7.02	[38]
_*14d*_ *ARD8–1*	8.04–8.05	Row number	umc1460–umc1562	8.04–8.05	[34]
_*9d*_*RSA8–1*, _*9d*_*TRV8–1*, _*14d*_*TRV8–1*	8.05	Ear length	umc1562–bnlg666	8.05	[34]
_*9d*_ *TRL9–1*	9.04	Row number	bnlg127–bnlg1209	9.03–9.04	[34]
		Leaf number	bnlg1208–umc1771	9.04	[38]
		Average internode length	bnlg1209–umc1771	9.04	[38]
		LTD	bnlg1209–umc2119	9.04	[36]
_*9d*_ *PSC10–1*	10.05	Kernel weight	umc1911–umc2043	10.04–10.05	[34]
_*14d*_ *PSC10–1*	10.06	Row number	umc1993–phi323152	10.05–10.06	[34]
		ARN in HN	umc2043–umc1061	10.05–10.06	[42]
		LTD	umc2122–phi323152	10.05–10.06	[36]

^a^For the abbreviations of the traits reported previously, five seedling root traits generated under low nitrogen (LN) or high nitrogen (HN) conditions (MARL, ARL, AARL, ARN, and LRL) [42] and three traits associated with water stress responses (LTD, RSFW, and RSDW) [36] are included. MARL, ARL, AARL, ARN, and LRL represent maximal axial root length, axial root length, average axial root length, axial root number, and lateral root length, respectively. LTD, RSFW, and RSDW represent leaf temperature differences, shoot fresh weight, and shoot dry weight, respectively

We first determined chromosomal regions with QTLs for seedling root traits and co-localized QTLs for grain yield and its three components, row number, ear length, and kernel weight [34]. The results showed co-localization of QTLs for seedling root traits and all four yield-related traits, with both traits controlled by seven chromosomal bin regions. As shown in Table 7, three QTLs for seedling root traits, one for grain yield, and one for row number co-localized at bin 1.03–1.04 on chromosome 1, and three QTLs for root traits co-localized with one QTL for ear length at bin 5.05. One QTL for root trait LRN, one for ear length, and one for grain yield co-localized at bin 7.02–7.03, and four QTLs for root traits and two for yield-related traits co-localized at bin 8.04–8.05 (Table 7). Two QTLs for the root trait PSC and two for yield-related traits co-localized at bin 10.05–10.06 (Table 7). Additionally, one QTL for root trait and one for grain yield-related trait co-localized at bin 4.03 and bin 9.04, respectively (Table 7). Taken together, fifteen of the seedling root trait QTLs identified in this study co-localized with eleven QTLs for grain yield-related traits. Furthermore, at least four QTLs that co-controlled seedling root traits and grain yield-related traits were located on each of the maize chromosomal bins 1.03–1.04, 5.05, 8.04–8.05, and 10.05–10.06.

We next analyzed chromosomal regions that contained QTLs for seedling root traits co-localized with QTLs for the content of the heavy metal As. Previous studies showed marked decreases in the As levels from the roots to the aerial tissues in both maize and rice [40, 43, 44], indicating the importance of root system in As absorption in cereal crops. Therefore, we compared the locations of QTLs for seedling root traits and those related to As content in the above-ground tissues in maize. Focusing on eleven QTLs for As content detected stably in two field locations [40], we found that seven QTLs for seedling root traits and one QTL for As content in the stem co-localized at bin 1.02–1.03, one QTL for root trait (RSA) and one for As content in the stem co-localized at bin 2.06–2.07, and one QTL for root trait (LRN) and one for As content in leaves co-localized at bin 7.02–7.03 (Table 7). It is noteworthy that among the three QTLs for As content that co-localized with QTLs for root traits, two controlled As content in the stem, which is the tissue closest to the root among all five above-ground tissues (kernel, axis, stem, bract, and leave) analyzed [40]. In total, nine QTLs for seedling root trait identified in this study co-localized with three QTLs for As content reported by Fu et al. [40] in three chromosomal regions.

We next examined chromosomal regions containing QTLs for seedling root traits co-localized with QTLs for plant height-related traits. Plant height-related traits are important for breeding because of their significant relationships to lodging resistance, planting density, and grain yield. QTLs for plant height and related traits, including internode number, average internode length, and leaf number, have been reported in the Zong3 × Yu87–1 RIL population by Tang et al. [38]. Therefore, we performed a comparative analysis of QTLs between seedling root traits and plant height-related traits. We found three co-localized regions on chromosome 1, among which seven QTLs for root traits and one QTL for internode number co-localized at bin 1.02, three QTLs for root traits and one QTL for internode number co-localized at bin 1.03, and one QTL for RTN (_*9d*_*RTN1–1*) and three QTLs for plant height-related traits co-localized at bin 1.07 (Table 7). Chromosomal regions controlling both seedling root traits and plant height-related traits also included bins 2.06–2.07, 3.04, 5.03, 5.05, 6.07, 7.02–7.03, and 9.04 (Table 7). Overall, twenty one QTLs for seedling root traits co-localized with seventeen of the total of twenty-seven QTLs for plant height-related traits [38] in ten chromosomal regions.

We also analyzed the co-localization of QTLs for seedling root traits and those for several traits generated under unfavorable conditions, including root traits under different supplies of nitrate [42], three physiological traits influenced by responses to water stress [36], and disease resistance [35]. This analysis showed that twenty QTLs for seedling root traits identified in this study co-localized with ten root trait QTLs reported under LN and HN conditions [42] on chromosomal bins 1.02, 1.03, 1.07, 2.06–2.07, 3.04, 5.05, 5.09, 6.07, and 10.06 (Table 7). In terms of QTLs for water stress-associated traits, i.e., leaf temperature differences (LTD) and two drought tolerance index traits, shoot fresh weight (SFW) and shoot dry weight (SDW), seven previously reported QTLs localized to the same or proximal bin regions (bins 1.07, 2.07, 9.04, and 10.06) as the QTLs for seedling root traits identified in this study (Table 7). One QTL responsible for resistance to the fungal disease known as common smut [35] co-localized with a root trait QTL identified at bin 5.03 in this study (Table 7).

To summarize, among the chromosomal regions containing QTLs for seedling root traits co-localized with other QTLs for physiological- or stress-related traits in the Zong3 × Yu87–1 RIL population, eight regions (bins 1.02, 1.03, 1.07, 2.06–2.07, 5.05, 7.02–7.03, 9.04, and 10.06) warrant further investigation of the mechanism by which these QTLs exert co-control of three or four more traits in addition to seedling root traits.

## Discussion

### The RIL population used in this study

Among the 508 individual maize germplasms collected globally, the inbred lines Zong3 and Yu87–1 display marked genetic divergence [41]. Moreover, the Zong3 × Yu87–1 combination, known as the Chinese hybrid Yuyu22, exhibits great heterotic performance in terms of a range of traits from seed germination and seedling growth, to crop yield [34, 38, 45–49]. The genetic mechanisms responsible for the different traits in the Zong3 × Yu87–1 RIL population have been intensively investigated [34–38, 40–42], and the F1 hybrids of the RILs, known as an “immortalized F2 population”, have been analyzed for the genetic basis of grain yield heterosis [37, 50, 51]. Based on the relatively clear genetic basis of multiple traits and the phenotypic variation we observed in seedling root traits (Table 2; Fig. 1), the Zong3 × Yu87–1 RIL population was selected as an ideal material for the analysis of the genetic architecture of root traits and molecular connections between seedling root and other traits.

### Genetic components responsible for maize seedling root traits

The genetic mechanism underlying the generation of different maize root traits has become a recent focus of research [21–27], and multiple QTLs that regulate different root traits in different linkage populations have been identified. Our study showed that the variations in eight seedling root traits identified across three time-points after germination in the Zong3 × Yu87–1 RIL are controlled by thirty-six QTLs. Each QTL accounted for between 8.9 and 23.2% of the phenotypic variation in seedling root traits, which is similar to the size of the effects of root trait QTLs reported in the B73 × Ki3 RIL population (5.5–23.8%) [23]. Most of these QTLs (86%) individually explained ≥10% of the seedling root trait variation. A number of root trait QTLs identified in this study are in good accordance with those in previous studies. A recent meta-analysis of QTLs for maize root length summarized putative QTL clusters based on the results of fifteen studies covering nine mapping populations [52]. Interestingly, among the four clusters listed as special noteworthy loci [52], two clusters, Ax-2 (at bin 1.07) and Ax-15 (at bin 7.03), overlapped with or were localized very close to the QTLs _*9d*_*TRN1–1* (at bin 1.07) and _*9d*_*LRN7–1* (at bin 7.02–7.03) identified in this study. In accordance with the reported role of bin 1.07 in the control of root number at different developmental stages [52], we show the involvement of this region (QTL _*9d*_*TRN1–1*) in controlling the total root number in seedlings at 9 dag (Table 6). Among the total twenty-three chromosomal bins identified here (Table 6), twelve regions were also detected in another study of root trait QTLs in a different Chinese RIL population [24], despite the differences in seedling age and cultivation conditions between the two studies. Among the twelve consensus regions, five chromosomal bins (bins 2.06, 2.09, 3.02, 5.05, and 9.04) were found to control at least three different root traits in two studies. For example, bin 5.05 controls four overall root traits in seedlings of a similar age. Specifically, three QTLs (_*14d*_*PRL5–1*, _*14d*_*RSA5–1*, and _*14d*_*TRL5–1*) for PRL, RSA, and TRL identified in seedlings at 14 dag in our study (Table 6) and one previously reported QTL for the ratio of total root dry weight to total shoot dry weight ratio (RSR) in 2-week-old seedlings [24] were identified. The chromosomal regions detected in both our research and previous studies may play an important role in the phenotypic variation in root traits under different genetic backgrounds and should be considered a priority for MAS studies to improve the target trait(s).

Compared to most other root trait QTL studies, one advantage of the current study is that we mapped root trait QTLs at three different time-points after germination. As a result, we were able to compare the locations of these QTLs not only at each time-point, but also between different time-points. For the QTLs identified at the same time-point, we detected co-localizations at each of the time-points, with a total of eight co-localized QTL regions: two regions at 4 dag, two at 9 dag, and four at 14 dag (Fig. 2; Table 6). It is intriguing to note that root traits controlled by each co-localized QTL region were correlated strongly or moderately to each other (*P* < 0.01). This finding may explain, at least partially, the phenomenon of root trait QTL overlap observed at each time-point. Furthermore, these findings may indicate the presence of a single gene exerting a strong effect or multiple tightly-linked genes with pleiotropic effects underlying each co-localized QTL region controlling root traits in the Zong3 × Yu87–1 RIL population.

Most root traits were significantly correlated at the three time-points (Table 4), although this may indicate the continuity and accumulation of each root trait over time. In contrast, we found no QTL co-localizations for root traits (either the same or different) among the three time-points (Fig. 2; Table 6). In combination, these findings suggest that the seedling root traits of the Zong3 × Yu87–1 RIL population are regulated by different sets of chromosomal regions or genes during the development of the root system.

The absence of co-localization of root trait QTLs among the three time-points (4, 9, and 14 dag) found in this study is inconsistent with a previous report of clustering of root trait QTLs at 4, 6 and 8 d after planting (dap) [23]. The divergence between the two studies may be attributable to the differences in root phenotyping, with our second time-point (9 dag) actually being later in the development than the third or last time-point (8 dap) in the previously reported study. Furthermore, the three time-points used in our study, cover much longer time intervals (5 and 10 days) than those in the previously reported study (2 and 4 days). It is possible that such short time intervals may not be long enough to result in the QTL location differences; therefore, multiple QTL clusters were found among the three time-points selected [23]. In contrast, it can be speculated that the longer time intervals between the time-points used in our study are sufficient to reflect the QTL location differences, and no QTL co-localizations were detected as a consequence (Fig. 2). Future investigation of more dense time-points is required to fully elucidate the developmental regulation of root traits. Alternative explanations for the divergence between our study and the previous report may include differences in plant cultivation methods, mapping populations, and the root traits investigated.

### Genetic association of maize seedling root traits with other traits

Although the significance of a good root system in maize has long been recognized [11, 25], the genetic association of the maize root system with other traits remains to be established. Chromosomal regions affecting both root traits and grain yield were investigated using QTL data from different populations more than 10 years ago [21, 25]. A more recent study evaluated the genetic relationship between root system architecture and the efficiency of nitrogen use [27]. In the current study, we surveyed the genetic association of seedling root traits with six other agronomic traits in one RIL population. We found that 41.7%, 25%, and 58.3% of the seedling root trait QTLs co-localized with QTLs for grain yield-related traits, QTLs for As content in above-ground tissues, and QTLs for plant height-related traits, respectively. We also found that 55.6% of the root trait QTLs we identified under normal conditions co-localized with the root trait QTLs detected under hydroponic LN or HN conditions [42]. Furthermore, among the nine QTLs for water stress-associated traits reported previously [36], seven co-localized with four root trait QTLs identified in our study. Overall, we found that a subgroup of seedling root trait QTLs overlapped with the QTLs for other traits generated under normal or unfavorable conditions, although the percentage of QTL overlapping in our study was not as high as the reported overlap (approximately 70%) between nitrogen-efficiency QTLs and those for root system architecture [27]. Reasons for the relatively small overlap between the QTLs for root traits and other traits found in our study may be related to the number of QTLs for each trait, where a small number of QTLs for a single trait would lower the overall percentage of overlapping QTLs, as well as differences in plant ages, growth environmental factors, and treatment conditions. In summary, our findings provide genetic evidence of the association of seedling root traits with several other traits in the Zong3 × Yu87–1 RIL population.

### Chromosomal regions valuable for root trait-based genetic selection to improve maize yield and other target traits

One of the main purposes of a maize root trait QTL study is to elucidate the genetic basis of development and identify important QTLs or chromosomal regions for use in selective breeding programs. Compared with the information for root trait QTLs alone, chromosomal regions controlling both root traits and other traits may provide a comprehensive platform for maize germplasm improvement. MAS is an efficient way to improve target trait(s) based on the QTLs detected, and it has been successfully exploited in maize breeding for several traits [53–55]. In this study, we identified chromosomal regions containing QTLs for seedling root traits co-localized with those for other traits. These were located in eight chromosomal bins (bins 1.02, 1.03, 1.07, 2.06–2.07, 5.05, 7.02–7.03, 9.04, and 10.06), each of which control three or four more different traits in addition to seedling root traits in the Zong3 × Yu87–1 RIL population. Some of these regions, such as bins 1.02, 1.03, 1.07, 7.02–7.03, were also found to be responsible for both root traits and grain yield in other populations [25]. The loci identified in this study may be good candidates for MAS to improve root traits as well as other traits of interest in parallel. Decreasing the co-localized chromosome intervals that controlled both root traits and other traits to increase the linkage reliability between the molecular markers and the phenotypes is the next step for effective MAS. A knowledge of the favorable alleles (from either the same or different parents) of the co-localized QTL region that are associated with root trait and other trait(s) of interest is also required to develop effective MAS-based breeding programs.

## Conclusion

In this study, we mapped QTLs for maize seedling root traits at three time-points in a RIL population. We then analyzed the QTL locations for root traits at the same and different time-points and determined the common chromosomal regions responsible for seedling root traits and other traits on the same genetic background. We found that the thirty-six QTLs identified overlapped at, but not between, each of the three time points, which suggests that different groups of genomic loci are involved in the regulation of seedling root traits in the Zong3 × Yu87–1 RIL population. Furthermore, through our analysis of the co-localizations of QTLs for both seedling root traits and six other traits, we have demonstrated the genetic association of seedling root traits with other agronomic traits, including grain yield in adult plants, in this RIL population. On the basis of previous studies, we have further clarified the genetic value of the Zong3 × Yu87–1 RIL population and improved our understanding of the genetic association of seedling root traits with other agronomic traits. Knowledge of the common chromosomal regions will enable breeders to improve maize performance in terms of root traits and other traits in parallel.

## Methods

### Plant materials and growth conditions

In this study, two maize parental lines, Zong3 and Yu87–1, and the F10 generation of the RIL population derived from these lines were used as genetic material for root trait analysis. Zong3 originates from a synthetic population of Chinese domestic germplasm, and Yu87–1 is selected from an exotic germplasm [35, 38]. The RIL population was constructed using the single-seed descent method, with Zong3 as the female parent and Yu87–1 as the male parent in the F1 generation [34]. For the parental lines, 15 seeds were tested at each time-point. For the Zong3 × Yu87–1 RIL population, 104 lines out of the 190 individual pools used to construct the high-density bin map [37] were tested. In each experiment, 6 seeds per RIL line at each of the indicated time-points were examined simultaneously at two locations. Two experimental replicates were conducted.

The maize seeds were surface sterilized with 10% (*v*/v) hydrogen peroxide for 40 min, rinsed three times with sterile water, and wrapped with a moist filter paper in a petri dish. The plates were then incubated in dark at 28°C for approximately 1.5 d for imbibition and seed germination, which was scored according the protrusion of a radicle (approximately 1 mm). Subsequently, uniformly germinated seeds were selected and sown in paper cups (top diameter × bottom diameter × height: 90 mm × 57 mm × 132 mm) with drainage holes (4–5) manually punched into the bottom. The cups were filled with clean quartz sand (grain size: 25–50 mesh) pretreated with 0.1% (v/v) HCl (12 M) overnight and rinsed five times with water before use. Two germinated seeds were planted in each cup, approximately 1 cm below the surface of the sand and then incubated in controlled growth chambers (day/night temperature 28°C/25°C with a 16-h light/8-h dark photoperiod). Full-strength Hoagland solution [56] was added to the samples once per day until sample collection.

### Root trait phenotyping

The maize seedlings were harvested for root trait analysis at 4 dag, 9 dag, and 14 dag. Briefly, the samples were carefully removed at the indicated time-points and rinsed with water to remove residual sand. After the PRL measurement and PSC scoring, each root system was cut from the shoot, and then the primary, seminal, and crown roots were separated before storage at 4°C. For phenotypic imaging of the root system, each sample was submerged in water to separate the roots and scanned under transmitted light using a Scanwise scanner (Amersham, UMAX). The images were then analyzed for the RTN, TRL, RSA, TRV, and ARD root traits using WinRHIZO software (Pro 2004b, Regent Instruments). LRN was calculated by subtraction of PSC from RTN.

### Root trait analysis

Differences in root traits between the two maize parental lines were analyzed by *t*-test using Excel software. For each root trait in the RIL family, the broad-sense heritability (*h*^*2*^) was calculated using SAS software (SAS Institute Inc., NC, USA) as described by Knapp et al., [57], and the distribution of each trait was analyzed using SPSS statistical software (SPSS, Inc., IL, USA). The Pearson correlation coefficients among traits in the RIL family were analyzed using SPSS statistical software.

### QTL mapping

A high-density genetic linkage map has been established for the Zong3 × Yu87–1 RIL population by single nucleotide polymorphism (SNP) genotyping of 190 lines using maize SNP50 chip [44]. In brief, 18,840 polymorphic markers between two parental lines were allocated to 3184 bins, and a bin-based genetic linkage map covering 2657.9 cM in total and corresponding to a physical map region of 2046.3 Mb of the maize B73 reference sequence was generated [37]. The average genetic and physical distances between adjacent bins were 0.84 cM and 0.64 Mb, respectively [37]. This genetic linkage map was used for QTL mapping of root traits in this study.

QTLs for seedling root traits were detected using Windows QTL Cartographer (version 2.5, model 6; [58]). The composite interval mapping (CIM) method [59] was used to scan the whole maize genome for QTLs, with forward stepwise regression as covariates and a setting of 10-cM window size and 0.5-cM scanning interval between markers. Putative QTLs for each trait were identified according to the threshold of the logarithm of odds (LOD) value, which was obtained via permutation test (1000 times) at a significance level of *P* < 0.05 [60]. The confidence interval of the QTL was defined as a 1.0-LOD reduction in the distance from the peak marker [61].

## Additional files

Additional file 1: **Figure S1.** Representative scanned image of Yu87–1 root system in a seedling at 9 dag. The primary root and all the seminal and crown roots of each seedling were cut from the shoot prior to scanning, and a representative image is shown. (PDF 20 kb)

Additional file 2: **Table S1.** Root trait data of the parental lines at the three time points. (XLSX 27 kb)

Additional file 3: **Table S2.** Root trait data of the RIL population at the three time points. (XLSX 135 kb)

Additional file 4: **Figure S2.** Frequency distributions of seedling root traits in the RIL population. The results of eight seedling root traits across three time-points are shown, with data from 4 dag shown in blue (A), data from 9 dag shown in yellow (B), and data from 14 dag shown in light green (C). (PDF 230 kb)

Additional file 5: **Table S3.** Statistical analysis of results (skewness and kurtosis) in relation to frequency distribution of seedling root traits in the RIL population. (PDF 19 kb)

## Abbreviations

AARL: Average axial root length
ARD: Average root diameter
ARL: Axial root length
ARN: Axial root number
As: Arsenic
cM: centimorgan
dag: days after germination
HN: High nitrogen
LN: Low nitrogen
LOD: Logarithm of odds
LRL: Lateral root length
LRN: Lateral root number
LTD: Leaf temperature differences
MARL: Maximal axial root length
MAS: Marker-assisted selection
PRL: Primary root length
PSC: Total number of the primary, seminal, and crown roots
QTL: Quantitative trait locus
RIL: Recombinant inbred line
RSA: Total root surface area
RSDW: Shoot dry weight
RSFW: Shoot fresh weight
RTN: Total root tip number
SNP: Single nucleotide polymorphism
SSR: Simple sequence repeat
TRL: Total root length
TRV: Total root volume
