# Supplementary material for: Genetic analysis of seedling root traits reveals the association of root trait with other agronomic traits in maize
Source: BMC Plant Biol. 2018 Aug 15;18:171. doi: 10.1186/s12870-018-1383-5 (PMC6094888; doi:10.1186/s12870-018-1383-5)
Supplement: Supplementary file 1 — Figure S1. Representative scanned image of Yu87–1 root system in a seedling at 9 dag. The primary root and all the seminal and crown roots of each seedling were cut from the shoot prior to scanning, and a representative image is shown. (PDF 20 kb) [file 12870_2018_1383_MOESM1_ESM.pdf]

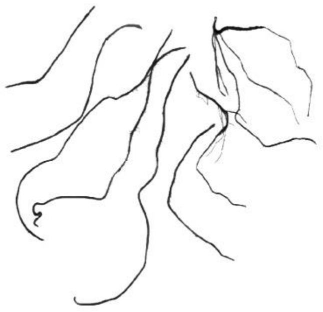

Yu87-1

Figure S1. Scanned image of Yu87-1 root system in a seedling 9 dag. The primary root and all the seminal and crown roots of each seedling were cut from the shoot prior to the scanning, and a representative image is shown.
