# Supplementary material for: Genetic analysis of seedling root traits reveals the association of root trait with other agronomic traits in maize
Source: BMC Plant Biol. 2018 Aug 15;18:171. doi: 10.1186/s12870-018-1383-5 (PMC6094888; doi:10.1186/s12870-018-1383-5)
Supplement: Supplementary file 4 — Figure S2. Frequency distributions of seedling root traits in the RIL population. The results of eight seedling root traits across three time-points are shown, with data from 4 dag shown in blue (A), data from 9 dag shown in yellow (B), and data from 14 dag shown in light green (C). (PDF 230 kb) [file 12870_2018_1383_MOESM4_ESM.pdf]

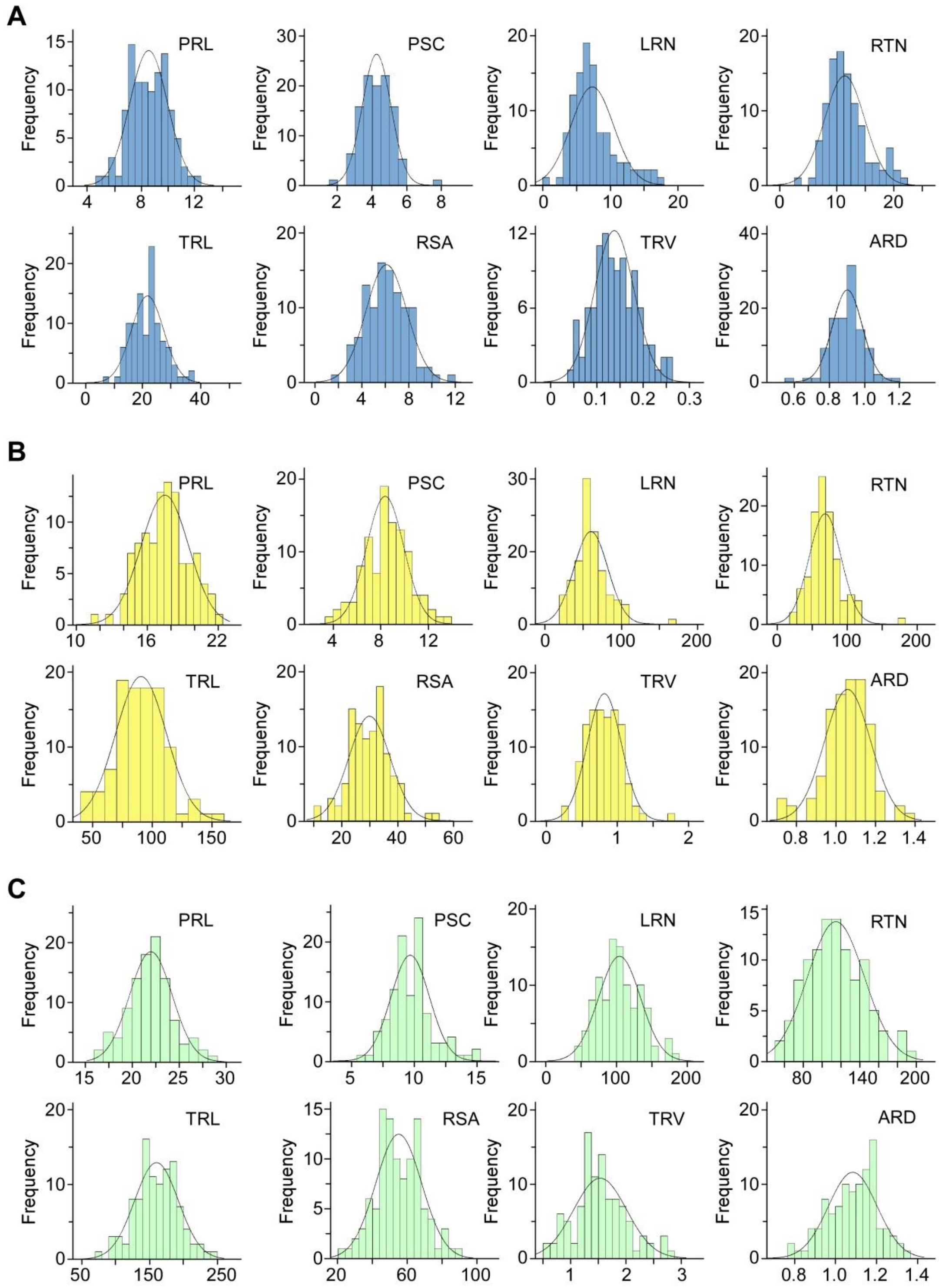

Figure S2. Frequency distributions of seedling root traits in the RIL population. The results of 8 seedling root traits across 3 time points are shown, with data from 4 dag showing in blue (A), data from 9 dag showing in yellow (B), and data from 14 dag showing in light green (C).
