# Supplementary material for: Genetic analysis of seedling root traits reveals the association of root trait with other agronomic traits in maize
Source: BMC Plant Biol. 2018 Aug 15;18:171. doi: 10.1186/s12870-018-1383-5 (PMC6094888; doi:10.1186/s12870-018-1383-5)
Supplement: Supplementary file 5 — Table S3. Statistical analysis of results (skewness and kurtosis) in relation to frequency distribution of seedling root traits in the RIL population. (PDF 19 kb) [file 12870_2018_1383_MOESM5_ESM.pdf]

Table S3. Statistical results (skewness and kurtosis) in relation to frequency distribution of seedling root traits in the RIL population.

**Root trait**	**4 dag**		**9 dag**		**14 dag**	
	Skewness	Kurtosis	Skewness	Kurtosis	Skewness	Kurtosis
PRL	0.024	-0.319	-0.062	-0.217	0.177	0.177
PSC	0.385	*1.327*	-0.042	0.108	0.584	0.754
LRN	0.978	0.760	*1.315*	*4.419*	0.316	-0.098
RTN	0.829	0.493	*1.236*	*4.101*	0.319	-0.107
TRL	0.323	0.234	0.229	0.644	0.035	0.029
RSA	0.301	0.169	0.260	0.865	0.139	0.048
TRV	0.236	-0.419	0.405	0.907	0.411	0.165
ARD	-0.215	*1.950*	-0.466	1.022	-0.323	-0.341

Statistical results of skewness and kurtosis were analyzed for seedling root traits in the RIL population, and values bigger than 1 are highlighted in italics.
